# Non-suicidal Self-Injury in the Context of Psoriasis, Post-traumatic Stress Disorder, and Acute Polysubstance Intoxication

**DOI:** 10.7759/cureus.112531

**Published:** 2026-07-12

**Authors:** Isabelle Csete, Arrionna Sackett, Edwin Meresh

**Affiliations:** 1 Psychiatry and Behavioral Sciences, Loyola University Medical Center, Maywood, USA

**Keywords:** addiction, chronic pain, dermatologic self-harm, non-suicidal self-injury, polysubstance intoxication, post-traumatic stress disorder, psoriasis, stimulant intoxication, trauma informed care

## Abstract

Non-suicidal self-injury (NSSI) during acute intoxication has been reported in association with stimulant and polysubstance use. Chronic somatic pain and trauma exposure may further increase vulnerability to impulsive self-injury. However, the intersection of inflammatory skin disease, addiction, and trauma-related pathology remains underdescribed in the clinical literature. We describe an unusual case of a hospitalized patient with psoriasis, post-traumatic stress disorder, and polysubstance use disorder who intentionally ignited psoriatic plaques during acute intoxication with cocaine, amphetamines, cannabis, benzodiazepines, and alcohol. The patient's behavior appeared to be motivated by overwhelming pruritus and pain, compounded by intoxication-related disinhibition and trauma-related emotional dysregulation. This case highlights pain-relief-motivated NSSI as a clinically important yet underrecognized phenomenon in addiction care. Chronic somatic illness, trauma history, and stimulant intoxication may converge to produce severe self-injury in the absence of suicidal intent. Addiction treatment models should incorporate assessment of dermatologic pain, trauma exposure, and psychosocial instability to reduce the risk of recurrent NSSI.

## Introduction

Non-suicidal self-injury (NSSI) is defined as deliberate, self-inflicted injury without suicidal intent and is commonly associated with emotional dysregulation, a history of trauma exposure, dissociation, and altered pain perception. Acute polysubstance intoxication is a common and dangerous precipitant of NSSI. NSSI involving dermatologic disease, particularly psoriatic lesions, highlights an important intersection of trauma, somatic pain, and substance use disorder, with the potential for significant psychiatric and medical morbidity.

Psoriasis is a chronic, immune-mediated inflammatory skin disease affecting approximately 2-3% of the global population and has a high prevalence of associated psychiatric comorbidities, including major depressive disorder, anxiety disorders, substance use disorders, suicidal ideation, and self-harm [1]. Clinically, psoriasis manifests as erythematous, scaly, and itchy plaques, although symptom severity varies considerably, and some patients experience minimal physical discomfort beyond visible skin lesions. Among symptomatic individuals, many patients describe cutaneous pain as burning, stinging, or raw. As a chronic inflammatory disease with systemic manifestations, psoriasis imposes a substantial pain burden that is associated with greater disease severity, psychological distress, reduced quality of life, and an increased risk of emotional impairment [2,3]. Population-level data from the United Kingdom demonstrate a 15% increased risk of self-harm among individuals with psoriasis compared with the general population, independent of disease severity or treatment status [4]. These findings make clear the emotional and cognitive sequelae of living with a painful and visibly stigmatizing condition.

Evidence further suggests a bidirectional relationship between psoriasis and addiction. A Swedish population-based cohort study found that individuals with psoriasis had a 1.4-fold increased risk of addiction, with alcohol use disorder being the most prevalent [5]. Notably, there was also a 4.3-fold increase in new substance use disorders following the onset of psoriasis [5]. These findings suggest that chronic somatic illness may contribute to the development of a substance use disorder as a maladaptive coping strategy, particularly among individuals with preexisting psychiatric vulnerabilities.

Addiction-related self-harm is clinically significant yet frequently underrecognized in acute care settings. Epidemiologic data from a Canadian emergency department registry found that 18.5% of acute cocaine-related presentations involved self-harm, underscoring the elevated risk of self-injurious behavior during stimulant intoxication [6]. Case reports similarly describe individuals engaging in cutting, burning, and other methods of NSSI while intoxicated with stimulants, often precipitated by emotional distress, trauma reactivity, paranoia, or psychosis [7].

Beyond behavioral disinhibition, stimulant intoxication may also contribute to self-injury through dermatologic and perceptual effects. Cocaine and amphetamines are associated with formication ("cocaine bugs"), pruritus, compulsive skin picking, and delusional parasitosis, symptoms that may intensify urges to scratch, excoriate, or even burn the skin [8,9].

Despite these overlapping risk domains, there remains limited clinical literature documenting chronic inflammatory disease, active substance use disorder, and psychiatric instability compounding to produce severe NSSI. This case report presents a patient with psoriasis, post-traumatic stress disorder (PTSD), and substance use disorders who intentionally ignited psoriatic plaques during an episode of polysubstance intoxication. The behavior occurred in the absence of suicidal intent and appeared to arise from a combination of intoxication-related disinhibition, psychiatric vulnerability, and attempts to modulate and quell severe psoriatic pain. This case highlights the importance of recognizing severe somatic pain as a potential driver of NSSI and underscores the need for integrated dermatologic, psychiatric, and addiction-focused assessment in patients with complex comorbidities.

## Case presentation

A 34-year-old male presented to the Loyola University Medical Center emergency department following transfer from an outside hospital for bilateral partial-thickness burns of the lower extremities covering approximately 4% total body surface area (Figure 1). 

**Figure 1:**
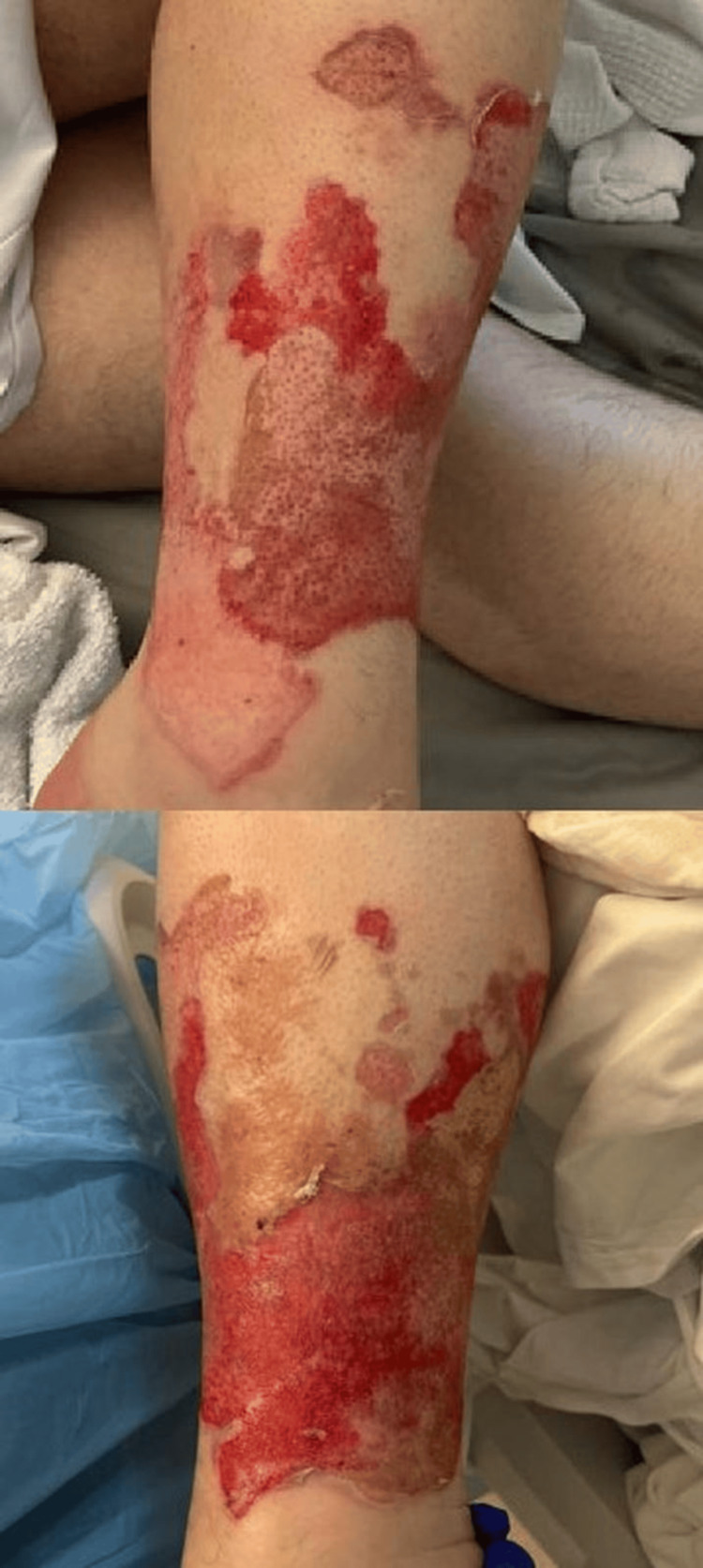
Bilateral partial-thickness burns of the lower extremities following ignition of psoriatic plaques.

At the time of injury, the patient was acutely intoxicated with cocaine, amphetamines, cannabis, benzodiazepines, and alcohol, confirmed by urine toxicology reports. The patient reported severe distress related to persistent itching and pain from the psoriatic plaques, which preceded the act of pouring gasoline on the affected sites and igniting them. The patient described his behavior as an attempt to relieve overwhelming somatic pain and discomfort.

The consultation-liaison psychiatry service was engaged to assess the need for inpatient placement due to concerns about possible suicidal intent and self-harm. During evaluation, the patient denied suicidal ideation and expressed a strong will to live, citing close family relationships as a significant protective factor. He explained that during intoxication, the pain from his psoriasis became intolerable and that the fire was intended as a desperate analgesic measure. Psychiatry concluded that the self-injury represented impulsive, intoxication-driven NSSI rather than a suicide attempt.

Further psychiatric assessment revealed a previously undiagnosed history of PTSD. The patient reported involvement in two significant motor vehicle collisions: one in 2016 as a passenger while intoxicated, and the other in 2019 as a sober driver whose vehicle was struck by a truck. Since the first accident, he has endorsed recurrent nightmares, intrusive flashbacks, hypervigilance, and avoidance behaviors. He also reported adverse childhood experiences, including physical abuse and exposure to parental substance use disorder, as contributing to early substance experimentation.

During interviews, the patient demonstrated mood lability with periods of animation alternating with tearfulness and constricted affect. Thought processes were intermittently tangential, with erratic shifts in conversation, suggestive of impaired executive control, likely exacerbated by his chronic stimulant use and acute intoxication. These emotional fluctuations paralleled the instability present in his daily life. Although previously employed in various roles, he was currently unemployed, aside from performing in a rock band and teaching guitar lessons, activities he described as meaningful. He expressed fear and concern that returning to his usual lifestyle and environment would increase his risk of substance use. While he demonstrated notable insight into the relationship between his environment and substance use disorder, he also acknowledged his difficulty exercising sound judgment in those moments. Further psychosocial assessment revealed a lack of stable housing, raising concern for relapse risk and safety following discharge.

Pharmacological treatment was initiated during hospitalization, including 1 mg of prazosin nightly for PTSD-related nightmares and 150 mg of bupropion extended-release daily for mood symptoms and stimulant withdrawal-related dysphoria. Burn wounds healed with supportive care.

Given the complexity of his psychiatric presentation, inpatient substance use and trauma-focused psychotherapy treatment were recommended. However, due to the patient's stated preferences, unsatisfactory social support, and lack of insurance coverage, he elected to pursue outpatient psychiatric follow-up with referrals to dermatology, addiction-focused group therapy, and trauma-focused psychotherapy.

## Discussion

This case illustrates an atypical and clinically significant presentation of chronic psoriatic pain and pruritus driving pain-relief-motivated NSSI in a hospitalized patient with unresolved trauma and polysubstance use disorder. Unlike most reported stimulant-associated self-harm cases driven by distress, psychosis, or trauma reactivity, this patient's behavior appeared primarily aimed at relieving intolerable pruritus and somatic pain.

A key feature of this unique presentation of NSSI is the dominant role of pruritus and cutaneous pain as behavioral drivers. Psoriatic itch and pain are strongly associated with psychiatric morbidity and impaired quality of life [10,11], but prior studies have largely viewed these symptoms as contributors to depression, anxiety, or suicidal ideation rather than as direct precipitants of self-injurious behavior. In contrast, this case suggests an escalation from severe somatic distress to intentional tissue-directed injury, expanding established psoriasis-psychological associations to include NSSI.

Psoriasis is also associated with increased substance use disorders and addictive behaviors [12,13], consistent with self-medication frameworks in which substances are used to regulate distress states. However, previous descriptions of this model focus on substance use for affect regulation rather than direct somatic self-injury [14]. This case therefore extends the self-medication hypothesis by suggesting that, during periods of marked intoxication and distress, maladaptive attempts at symptom relief may extend beyond substance ingestion to include tissue-directed harm.

Polysubstance intoxication likely played a key role in precipitating this behavior by reducing inhibitory control while amplifying somatic and perceptual distress. Stimulant-related dermatologic phenomena, including formication, excoriation, and delusional parasitosis, are well documented (Table 1) [8,9], and prior reports of stimulant-associated self-harm predominantly describe psychosis- or paranoia-driven injury. However, unlike these previously described mechanisms, the present case lacked fixed psychotic beliefs or infestation delusions, indicating a distinct clinical phenotype in which self-injury was motivated by perceived somatic relief rather than perceptual distortion.

**Table 1:** Dermatologic manifestations associated with cocaine and amphetamine use. Table credits: Arrionna Sackett and Isabelle Csete. Information derived from references [8,9]. OCD: obsessive-compulsive disorder.

Manifestation	Description	Psychiatric Association
Formication (“cocaine bugs”)	Tactile hallucinations of insects crawling on or under the skin	Linked to stimulant-induced psychosis
Delusional parasitosis	Fixed, false belief of skin infestation, often associated with excoriation	Linked to paranoia secondary to stimulant-induced psychosis
Cocaine-induced pruritus	Generalized itching, related to vasoconstriction or neurotoxicity induced by cocaine	May lead to excoriation and self-inflicted injury
Excoriations	Repeated picking of the skin, leading to lesions, sores, wounds, and scarring	Can occur with anxiety, OCD, and stimulant use

Acute stimulant intoxication and polysubstance dependence are also associated with increased impulsivity, impaired executive control, heightened pain perception, and enhanced emotional reactivity. Polysubstance-using populations demonstrate higher levels of childhood trauma exposure, aggression, psychosis-related personality traits, and self-injurious behavior compared with monosubstance users. Additionally, childhood maltreatment independently increases the risk of NSSI, PTSD, and affective dysregulation in substance-using populations [15]. Together, these findings support a cumulative vulnerability model in which baseline trauma burden and trait-level dysregulation interact with intoxication-related disinhibition, amplifying the risk of severe self-injurious behavior.

This case extends existing stimulant-associated NSSI literature by suggesting the possibility of a pain-relief-driven clinical phenotype that has not been clearly characterized in prior studies. Given that this is a single case report, this concept should be considered hypothesis-generating and warrants further investigation before broader clinical conclusions can be drawn. Neurobiologically, overlapping dopaminergic and endogenous opioid pathways may link pruritus, addiction, and compulsive behaviors [16]. Within this framework, stimulant use may exacerbate both itch intensity and reward-driven dysregulation, creating a feed-forward loop of somatic distress and maladaptive coping that differs from more commonly described psychosis-mediated mechanisms.

From a mechanistic perspective, the endogenous opioid model of NSSI offers one potential framework for understanding pain-relief-motivated self-injury. This framework proposes that self-injury transiently activates endogenous opioid signaling, producing short-lived analgesia and negative reinforcement that maintains the behavioral cycle. In individuals with chronic pain and trauma histories, altered pain processing and heightened affective vulnerability further increase susceptibility to self-injury as a regulatory strategy [17]. In this case, burning psoriatic plaques represented an extreme attempt to interrupt intolerable pruritic and nociceptive signaling.

Collectively, these findings represent a clinically underrecognized intersection of inflammatory dermatologic disease with severe pruritus, polysubstance intoxication, and trauma-related emotional dysregulation, which together culminated in pain-relief-motivated NSSI. This combination is not explicitly characterized in the existing literature and may represent a distinct clinical presentation deserving further study, emphasizing a gap in current approaches to risk stratification and diagnostic framing. However, as a single case report, these observations cannot establish causality or generalizability and should instead be viewed as hypothesis-generating.

These findings also have implications for care. Evidence supports integrated treatment models combining trauma-focused psychotherapy and substance use interventions, with large effect sizes for both PTSD and substance-related outcomes [18]. Given the central role of severe pruritus and cutaneous pain in this presentation, coordinated care models that integrate dermatologic management with psychiatric and addiction treatment may further improve outcomes in complex patients [19]. Within this framework, clinical assessment should incorporate evaluation for somatic pain-driven self-injury, a mechanism not routinely captured in standard psychiatric or addiction screening but directly relevant to patients with severe pruritus or inflammatory skin disease.

## Conclusions

This case documents a unique presentation of pain-relief-motivated NSSI during acute polysubstance intoxication and highlights the importance of recognizing chronic somatic pain as a potential driver of dangerous self-injury in addiction care settings. It illustrates how stimulant intoxication, trauma-related vulnerability, and untreated inflammatory disease may converge to produce life-threatening behavior. These findings underscore the need for integrated, trauma-informed, addiction-focused care models that address the complex interplay between chronic illness, trauma, and substance use disorders. Clinically, addiction-focused care should incorporate assessment of somatic pain sources, particularly dermatologic conditions that may worsen during intoxication or withdrawal. Careful evaluation of trauma histories is essential, as unresolved trauma may reduce distress tolerance and impair judgment during intoxication. Additionally, social determinants such as unstable housing, limited insurance coverage, and inconsistent access to dermatologic care significantly influence treatment adherence and outcomes. Further studies are needed to determine whether similar presentations occur in broader patient populations and to better define the role of chronic somatic pain in NSSI among individuals with substance use disorders.
